# Trends in maternal and neonatal mortality in South Africa: a systematic review

**DOI:** 10.1186/s13643-019-0991-y

**Published:** 2019-03-27

**Authors:** Damian J. Damian, Bernard Njau, Ester Lisasi, Sia E. Msuya, Andrew Boulle

**Affiliations:** 1 School of Public Health and Family Medicine, University of Cape Town, Cape Town, South Africa; 2 Institute of Public Health, Kilimanjaro Christian Medical Centre, Moshi, Tanzania

**Keywords:** Maternal mortality, Neonatal mortality, Millennium Development Goals, South Africa

## Abstract

**Background:**

Measuring and monitoring progress towards Millennium Development Goals (MDG) 4 and 5 required valid and reliable estimates of maternal and child mortality. In South Africa, there are conflicting reports on the estimates of maternal and neonatal mortality, derived from both direct and indirect estimation techniques. This study aimed to systematically review the estimates made of maternal and neonatal mortality in the period from 1990 to 2015 in South Africa and determine trends over this period.

**Methods:**

Nationally-representative studies reporting on maternal and neonatal mortality in South Africa were included for synthesis. Literature search for eligible studies was conducted in five electronic databases: Medline, Africa-Wide Information, Scopus, Web of Science and CINAHL. Searches were restricted to articles written in English and presenting data covering the period between 1990 and 2015. Reference lists of retrieved articles were screened for additional publications, and grey literature was searched for relevant documents for the review. Three independent reviewers were involved in study selection, data extractions and achieving consensus.

**Results:**

In total, 969 studies were retrieved and 670 screened for eligibility yielding 25 studies reporting data on maternal mortality and 14 studies on neonatal mortality. Most of the studies had a low risk of bias. Estimates from the institutional reporting differed from the international metrics with wide uncertainty/confidence intervals. Moreover, modelled estimates were widely divergent from estimates obtained through empirical methods. In the last two decades, both maternal and neonatal mortality appear to have increased up to 2009, followed by a decrease, more pronounced in the care of maternal mortality.

**Conclusion:**

Estimates from both global metrics and institutional reporting, although widely divergent, indicate South Africa has not achieved MDG 4a and 5a goals but made a significant progress in reducing maternal and neonatal mortality. To obtain more accurate estimates, there is a need for applying additional estimation techniques which utilise available multiple data sources to correct for underreporting of these outcomes, perhaps the capture-recapture method.

**Systematic review registration:**

PROSPERO CRD42016042769

**Electronic supplementary material:**

The online version of this article (10.1186/s13643-019-0991-y) contains supplementary material, which is available to authorized users.

## Background

Monitoring progress towards MDG 4 and 5 (reducing child and maternal mortality between 1990 and 2015) required valid, reliable and internationally comparable estimates of maternal and child mortality in the country. Various methods for measuring and estimating maternal and child mortality have been developed, tested and widely used [1–8]. Estimating these outcomes in developing countries is challenging due to the lack of accurate, valid and reliable data [8–14].

Recent estimates from the United Nations Inter-agency Group for Child Mortality Estimation (UN-IGME) and Maternal Mortality Estimation Inter-agency Group (MMEIG) indicated that South Africa did not achieve the MDG 4a and 5b targets by 2015 (reducing by three quarters the maternal mortality ratio (MMR) and reducing by two thirds the under-five mortality rate in the period between 1990 and 2015, respectively) [9, 14]. Considering other African countries which did not meet MDG 4 and 5 targets, only South Africa had conflicting estimates of maternal and neonatal mortality reported by different sources with wide uncertainty intervals [9, 14–18].

South Africa is unusual among developing countries in that national facility-based mortality audits are carried out for maternal, perinatal and child deaths [19, 20]. Estimation of maternal and neonatal mortality in the country is often based on the vital registration, National Confidential Enquiry into Maternal Deaths (NCEMD) which records maternal deaths and the Perinatal Problem Identification Program (PPIP) which records stillbirths and neonatal deaths [19–22]. The CEMD data provides the maternal deaths from the routine surveillance of maternal deaths at a facility level whereas vital registration data derives deaths from the causes of deaths, as well as surveys and censuses provide maternal deaths from the collected pregnancy-related data at a household level. Nonetheless, Stats SA are the custodians of vital registration and the Department of Health are the custodians of the confidential enquiries.

The country provides unique opportunities to estimate these outcomes empirically, analytically or through modelling, by having multiple data sources with wide coverage [1–5, 21, 23, 24]. However, there are widely divergent estimates, wherein the two most frequently cited estimates are from institutional reporting and WHO metrics, which makes it difficult to both understand trends in these outcomes and to assess the successes or failures of interventions focusing on reducing maternal and child mortality in the country over the past decades. The reasons for divergent estimates between institutional reporting and WHO metrics, or among global metrics, can be explained by estimation approaches, sources and quality of data used [23, 25, 26].

Monitoring maternal and neonatal mortality in South Africa over the past two decades is of high importance given the introduction of Termination of Pregnancy Act in 1996 which has reduced the extent of abortion-related maternal morbidity and mortality as well as the context of high HIV prevalence and its associated mortality in women during pregnancy and childbirth [27, 28]. There has also been a massive uptake of HIV treatment and prevention of mother-to-child transmissions (PMTCT) of HIV, which currently stands at over 90% by some estimates [29, 30].

There have been limited attempts to review maternal and neonatal mortality estimates in South Africa to facilitate understanding of trends during the MDG period. This review is expected to provide the context for understanding inconsistencies in reported estimates of maternal and neonatal mortality by the institutional reporting and the global metrics by ascertaining estimation methods, data sources and quality, sampling methods and definitions used, to better inform comparisons across such estimates.

### Aim

This review aimed to synthesise estimates of maternal and neonatal mortality for the period 1990 to 2015 in South Africa and to determine temporal trends during this period.

## Methods

### Protocol and registration

The review protocol was registered with the PROSPERO database in 2016 with a registration number CRD42016042769 (http://www.crd.york.ac.uk/PROSPERO/display_record.php?ID=CRD42016042769) and has already been published [31]. The presentation and reporting of results in this review followed the systematic review reporting standard (PRISMA-P) [32]. To ensure transparency, a PRISMA flow chart was used and a table indicating all included studies was presented [33].

### Eligibility criteria

The population for eligible studies included pregnant women and neonates for ascertaining maternal and neonatal mortality, respectively. All studies that are nationally representative, reports providing national-level data (and trends thereof) and vital registration data were eligible for this review. Searches were restricted to studies written in English and being conducted in South Africa or which have used South African data, and multicentre studies including South Africa, reporting data covering the period 1990 to 2015. No restrictions on the date of publication were made in order to include articles reporting data from 1990 to 2015 which are published beyond 2015.

### Information sources

Separate searches for the two outcomes (maternal and neonatal mortality) were conducted in the following electronic databases: Medline, Africa-Wide Information, Scopus, Web of Science and CINAHL. The last search was carried out on 18 August 2017. No restrictions on the date of publication were made. Additional searches for conference abstracts and proceedings were made. Reference lists of retrieved articles were also screened for additional publications. Reports by the government or other agencies were included based on publications, and a number of data sources reported by them were included. Contacts with experts in the field of study were made to identify additional relevant articles.

### Search

The searches in the forementioned electronic databases were conducted from August 2016 to August 2017. All searches were restricted to articles written in English and reporting data covering the period from 1990 to 2015. In particular, the search strategy used in Medline database was as follows: ((“mothers”[MeSH Terms] OR “mothers”[All Fields] OR “maternal”[All Fields]) OR (“infant, newborn”[MeSH Terms] OR (“infant”[All Fields] AND “newborn”[All Fields]) OR “newborn infant”[All Fields] OR “neonatal”[All Fields])) AND ((“mortality”[Subheading] OR “mortality”[All Fields] OR “mortality”[MeSH Terms]) OR (“death”[MeSH Terms] OR “death”[All Fields])) AND (estimation[All Fields] OR estimates[All Fields]) AND (“South Africa”[Mesh] OR (“south africa”[MeSH Terms] OR (“south”[All Fields] AND “africa”[All Fields]) OR “south africa”[All Fields])) AND ((“1990/01/01”[PDAT]: “3000/12/31”[PDAT]) AND “humans”[MeSH Terms] AND English[lang]) Additional file 1.

### Study selection

Search outputs were managed in EndNote reference manager. Any duplicate records were removed before the screening process takes place. When the same article was captured in different journals or the same results were presented with different main authors, the most detailed publications were selected for review. Three independent reviewers were involved in the screening and selection of articles to be included in a quantitative (narrative) synthesis. This involved an assessment of articles based on titles and abstracts and full-text review using Covidence software (https://www.covidence.org/). For an article to be eligible for inclusion in the systematic review, two reviewers had to agree to include it. A third reviewer was consulted in case of any difference of opinion between the two reviewers. This followed when they failed to reach a consensus after a joint examination of the different views.

### Data collection process

Analysis of the full text was conducted for all eligible articles. Two authors extracted data independently using a pre-agreed data abstraction template. In the case of discrepancies in the extracted data between authors, consensus was sought before involving a third author for resolving the disparities. During the data extraction process, study authors/investigators were contacted to provide extra information when there were insufficient information/data reported in the article.

### Data items

The following information was extracted for eligible studies: first author’s name; year of publication; year of death (maternal and neonatal); number of pregnant women; number of live births; maternal deaths; neonatal deaths; definition of maternal death; definition of neonatal death; maternal mortality ratio/rate (if reported, and by year); neonatal mortality rate (if reported, and by year); sampling method; estimation method used; and an indicator variable whether the records are complete.

The main outcomes in this review were maternal and neonatal mortality. Maternal death/mortality was defined as the death of a woman while pregnant or within 42 days of termination of pregnancy, irrespective of the duration and site of the pregnancy, from any cause related to or aggravated by the pregnancy or its management but not from accidental or incidental causes [34]. Maternal mortality ratio (MMR) was defined as the number of maternal deaths per 100,000 live births.

Neonatal death/mortality was also defined as the death of live-born within the first 28 days of life. Neonatal mortality rate was defined as the number of infant deaths within the first 28 days of life per 1000 live births.

### Risk of bias in individual studies

Assessment of risk of bias was done at a study and outcome level. Two authors assessed the study quality based on the following quality assessment criteria: (1) definition of maternal mortality, (2) definition of neonatal deaths, (3) completeness of ascertainment of maternal and neonatal mortality, (4) completeness of ascertainment of live births, (5) sampling technique/design and (6) data quality. Studies were assessed based on each criterion and were rated as “high risk of bias” or “low risk of bias” accordingly. Studies rated as high risk of bias on any criterion were assigned an overall rating of high risk of bias while the overall rating of low risk of bias was only assigned in studies with low risk of bias in all criteria. For model-based estimations, risk of bias was assessed based on the input data used. Reports by the government and other agencies such as Stat SA, National Department of Health and WHO were assessed using similar criteria as empirical studies. Table 1 shows the assessment criteria of risk of bias in individual studies.

**Table 1:** Risk of bias assessment criteria for individual studies

No.	Criteria	Attributes	Risk of bias
1.	Definition of maternal mortality	❖ ICD-10 maternal death definition [85], or similar	Low
		❖ No or unclear definition provided	High
2.	Definition of neonatal mortality	❖ Death of live-born within the first 28 days of life, or similar	Low
		❖ No or unclear definition provided	High
3.	Completeness of ascertainment of maternal and neonatal deaths	❖ Prospective recording of mortality data ❖ Mixed methods cross-referencing facility records ❖ Demographic surveillance system with frequent rounds ❖ Survey based on recall of maternal or neonatal deaths ≤ 6 months previously	Low
		❖ Survey using direct or indirect sisterhood estimation methods ❖ Demographic surveillance system with infrequent rounds.	High
4.	Completeness of ascertainment of live births	❖ Prospective recording of births data ❖ Use of census < 5 years old for live births	Low
		❖ Use of census ≥ 5 years old for live births ❖ Live births data source not stated or unclear	High
5.	Sampling technique/design	❖ Census ❖ Vital registration ❖ Survey using nationally representative sample ❖ Systematic analysis involving the use of data collected from the above method(s)	Low
		❖ Design or sampling techniques not stated or unclear ❖ Provincial or sub-national sample used	High
6.	Data quality	❖ Data provide enough information for the study	Low
		❖ Insufficient data provided or unclear	High

### Summary measures

Data were presented as ratios for maternal mortality and rates for neonatal mortality with their corresponding confidence or uncertainty intervals.

### Synthesis of results

Data were entered and analysed using STATA software version 14.1 (Stata Corp, College Station, Texas). Data were presented as MMR or NMR in tables and graphs to depict trends over time. The reasons for study exclusions were clearly documented.

## Results

### Study selection

**Fig. 1:**
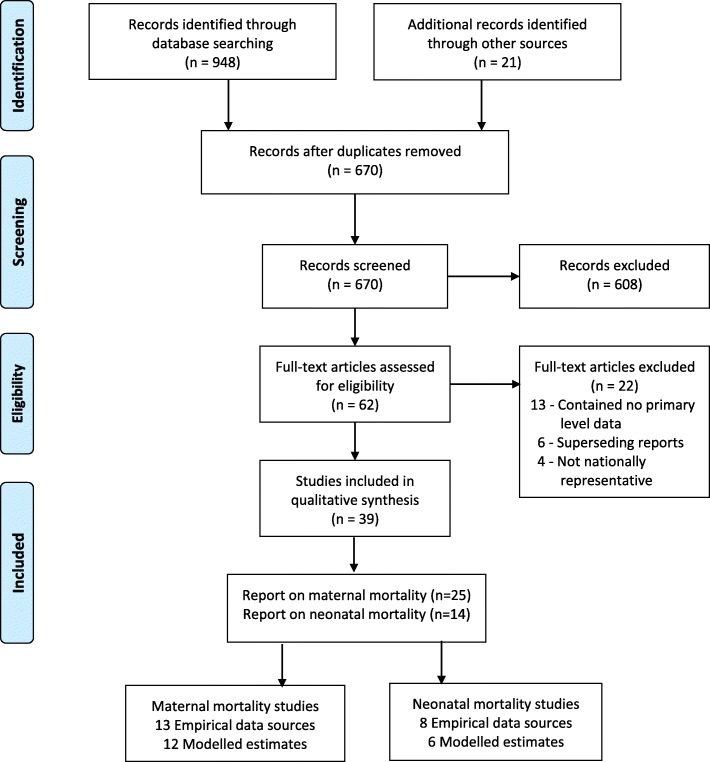
Flow diagram of study selection for inclusion in the qualitative synthesis

As presented in Fig. 1 below, a total of 948 studies were identified through the literature search and 21 additional studies were identified through screening of reference lists. After removing the duplicates, 670 studies were screened for eligibility. A total of 608 studies were excluded after screening the titles and abstracts as they reported irrelevant information. Sixty-two abstracts were shortlisted for full-text review and 39 studies met the inclusion criteria for analysis. Of studies included in the review, 25 reported data on maternal mortality [28, 35–55] and 14 on neonatal mortality [14, 23, 40, 45, 55–64].

### Characteristics of the included studies

#### Maternal mortality

Table 2 depicts the characteristics of studies reporting maternal mortality data. All studies were nationally representative presenting national level data covering a period between 1990 and 2015. Twelve studies estimated MMR though modelling [35–44, 65, 66] while 13 studies estimated MMR empirically [28, 45–55, 67]. Regarding the study design, 11 studies based on modelling [35–44, 66], seven active surveillance [28, 48, 49, 51, 53, 54, 67], three vital registration [45–47], two population-based household [55, 65] and a census [52]. The most common definitive data source was the Confidential Enquiry into Maternal Deaths (CEMD) (*n* = 7) [28, 48, 49, 51, 53, 54, 67] followed by the WHO models (*n* = 6) [35, 36, 39, 40, 42, 44] and vital registration (*n* = 3) [45–47].

**Table 2:** Study characteristics for maternal mortality data

No.	Author (year)	Duration covered	Data source/type	Definitive data source	Design	Estimation method
1.	Dorrington et al., 2016 [45]	2008–2013	Empirical data	Vital registration	Vital registration	Direct estimation
2.	WHO, 2015 [35]	1990–2015	Modelling	WHO	Modelling	Bayesian maternal mortality estimation model
3.	MDG/Stats SA, 2015 [46]	1998–2013	Empirical data	Vital registration	Vital registration	Direct estimation
4.	Dorrington et al., 2015 [47]	2011–2014	Empirical data	Vital registration	Vital registration	Direct estimation
5.	WHO, 2014 [36]	1990–2013	Modelling	WHO	Modelling	Multilevel-regression model
6.	Kassebaum et al., 2014 [37]	1990–2013	Modelling	IHME	Modelling	Cause of Death Ensemble model (CODEm)
7.	Department of Health, 2014 [48]	2005–2014	Empirical data	CEMD	Active surveillance	Direct estimation
8.	NCCEMD, 2014 [49]	2011–2013	Empirical data	CEMD	Active surveillance	Direct estimation
9.	Udjo, 2014 [38]	2001–2007	Modelling	Model A	Modelling	Growth Balance method + Relation Gompertz model
10.	Pattinson et al., 2013 [67]	1999–2012	Empirical data	CEMD	Active surveillance	Direct estimation
11.	WHO, 2012 [39]	1990–2010	Modelling	WHO	Modelling	Multilevel-regression model
12.	NCCEMD, 2012 [28]	2008–2010	Empirical data	CEMD	Active surveillance	Direct estimation
13.	Garenne, 2011 [65]	2007	Modelling	Model B	Population-based survey	Linear logistic model
14.	WHO, 2011 [40]	1990–2009	Modelling	WHO	Modelling	Bayesian maternal mortality estimation model
15.	Lozano et al., 2011 [41]	1990–2011	Modelling	IHME	Modelling	Cause of Death Ensemble model (CODEm)
16.	Stats SA, 2011 [50]	2008–2009	Empirical data	Vital registration	Vital registration/census	Direct estimation
17.	WHO, 2010 [42]	1990–2008	Modelling	WHO	Modelling	Multilevel-regression model
18.	Hogan et al., 2010 [43]	1980–2008	Modelling	IHME	Modelling	Generalised negative binomial regression
19.	NCCEMD, 2008 [51]	2005–2007	Empirical data	CEMD	Active surveillance	Direct estimation
20.	Garenne et al., 2008 [52]	2001	Census	Census	Census	Direct estimation
21.	Moodley, 2003 [53]	1999–2001	Empirical data	CEMD	Active surveillance	Direct estimation
22.	AbouZahr et al., 2001 [44]	2000	Modelling	WHO	Modelling	Robust regression
23.	Hill et al., 2001 [66]	1995	Modelling	Model C	Modelling	Robust regression
24.	Moodley, 2000 [54]	1999	Empirical data	CEMD	Active surveillance	Direct estimation
25.	SADHS, 1998 [55]	1992–1998	Empirical data	DHS	Population-based survey	Direct sisterhood

*CEMD* Confidential Enquiry to Maternal Deaths, *IHME* Institute for Health Metrics and Evaluation, *DHS* Demographic and Health Survey, *WHO* World Health Organization

#### Neonatal mortality

The study characteristics for neonatal mortality data are presented in Table 3 below. Eight studies used empirical data sources [23, 45, 55–60] whereas six studies based on modelling [14, 40, 61–64]. Regarding design, five studies were modelling/systematic analysis [14, 61, 62, 64], three vital registration [23, 45, 57], three population surveys [55, 56, 60] and two active surveillance [58, 59]. The most dominant definitive data sources for neonatal mortality estimates were vital registration [23, 45, 57] and population surveys [55, 56, 60], respectively.

**Table 3:** Study characteristics for neonatal mortality data

No.	Author (year)	Duration covered	Data source/type	Definitive data source	Design	Estimation method
1.	SADHS, 2016 [56]	2011–2016	Empirical data	DHS	Population-based survey	Direct estimation
2.	Dorrington et al., 2016 [45]	2012–2015	Empirical data	Vital registration	Vital registration	Direct estimation
3.	UNICEF, 2015 [14]	1990–2015	Modelling	UNICEF	Modelling	Bayesian hierarchical splines regression
4.	UNICEF, 2014 [61]	1990–2013	Modelling	UNICEF	Modelling	Bayesian hierarchical splines regression
5.	Dorrington et al., 2014 [57]	2009–2013	Empirical data	Vital registration	Vital registration	Direct estimation
6.	Pattinson et al., 2014 [23]	2012–2013	Empirical data	Vital registration	Vital registration	Direct estimation
7.	NaPeMMCO, 2014 [58]	2010–2013	Empirical data	PPIP	Active surveillance	Direct estimation
8.	WHO, 2011 [40]	1990–2009	Modelling	WHO	Modelling	Bayesian B-splines bias-adjusted model
9.	Oestergaard et al., 2011 [62]	1990–2009	Modelling	Model A	Modelling	Multilevel-regression model
10.	NaPeMMCO, 2011 [59]	1997–2008	Empirical data	PPIP	Active surveillance	Direct estimation
11.	Rajaratnam et al., 2010 [63]	1970–2010	Modelling	Model B	Modelling	Gaussian process regression
12.	SADHS, 2007 [60]	1998–2003	Empirical data	DHS	Population-based survey	Direct estimation
13.	Hyder et al., 2003 [64]	1995	Modelling	Model C	Modelling	UN projections
14.	SADHS, 1998 [55]	1988–1998	Empirical data	DHS	Population-based survey	Direct estimation

### Risk of bias within studies

Table 4 below presents the assessment of risk of bias of the individual studies. A total of 11 studies reporting maternal mortality data [28, 38, 49, 51–55, 65–67] and six studies reporting neonatal mortality data [23, 55, 56, 60, 62, 64] had overall high risk of bias. Among studies reporting maternal mortality data, three studies did not use the ICD-10 definition of maternal death [52, 65, 66], eight studies used data which were not population-representative [28, 38, 48, 49, 53–55, 67], one study used sisterhood estimation methods [55] and the sampling technique was unclear in one study [53]. Of seven studies reporting neonatal mortality data having an overall high risk of bias, four were not population-representative [23, 62, 64] and three were surveys based on recall of neonatal deaths more than 6 months previously [55, 56, 60].

**Table 4:** Risk of bias assessment in individual studies

No.	Author (year)	Definition	Ascertainment of deaths/live births	Sampling technique/design	Data quality	Overall risk of bias
Maternal mortality						
1.	Dorrington et al., 2016 [45]	Low	Low	Low	Low	Low
2.	WHO, 2015 [35]	Low	Low	Low	Low	Low
3.	MDG/Stats SA, 2015 [46]	Low	Low	Low	Low	Low
4.	Dorrington et al., 2015 [47]	Low	Low	Low	Low	Low
5.	WHO, 2014 [36]	Low	Low	Low	Low	Low
6.	Kassebaum et al., 2014 [37]	Low	Low	Low	Low	Low
7.	Department of Health, 2014 [48]	Low	High	Low	Low	High
8.	NCCEMD, 2014 [49]	Low	High	Low	Low	High
9.	Udjo, 2014 [38]	Low	Low	Low	Low	Low
10.	Pattinson et al., 2013 [67]	Low	High	Low	Low	High
11.	WHO, 2012 [39]	Low	Low	Low	Low	Low
12.	NCCEMD, 2012 [28]	Low	High	Low	Low	High
13.	Garenne, 2011 [65]	High	Low	Low	Low	High
14.	WHO, 2011 [40]	Low	Low	Low	Low	Low
15.	Lozano et al., 2011 [41]	Low	Low	Low	Low	Low
16.	Stats SA, 2011 [50]	Low	Low	Low	Low	Low
17.	WHO, 2010 [42]	Low	Low	Low	Low	Low
18.	Hogan et al., 2010 [43]	Low	Low	Low	Low	Low
19.	NCCEMD, 2008 [51]	Low	High	Low	Low	High
20.	Garenne et al., 2008 [52]	High	Low	Low	Low	High
21.	Moodley, 2003 [53]	Low	High	High	Low	High
22.	AbouZahr et al., 2001 [44]	Low	Low	Low	Low	Low
23.	Hill et al., 2001 [66]	High	Low	Low	Low	High
24.	Moodley, 2000 [54]	Low	High	Low	Low	High
25.	SADHS, 1998 [55]	Low	High	Low	Low	High
Neonatal mortality						
1.	SADHS, 2016 [56]	Low	High	Low	Low	High
2.	Dorrington et al., 2016 [45]	Low	Low	Low	Low	Low
3.	UNICEF, 2015 [14]	Low	Low	Low	Low	Low
4.	UNICEF, 2014 [61]	Low	Low	Low	Low	Low
5.	Dorrington et al., 2014 [57]	Low	Low	Low	Low	Low
6.	Pattinson et al., 2014 [23]	Low	High	Low	Low	High
7.	NaPeMMCO, 2014 [58]	Low	Low	Low	Low	Low
8.	WHO, 2011 [40]	Low	High	Low	Low	High
9.	Oestergaard et al., 2011 [62]	Low	High	Low	Low	High
10.	NaPeMMCO, 2011 [59]	Low	Low	Low	Low	Low
11.	Rajaratnam et al., 2010 [63]	Low	Low	Low	Low	Low
12.	SADHS, 2007 [60]	Low	High	Low	Low	High
13.	Hyder et al., 2003 [64]	Low	High	Low	Low	High
14.	SADHS, 1998 [55]	Low	High	Low	Low	High

### Results of individual studies and data synthesis

#### Maternal mortality

**Fig. 2:**
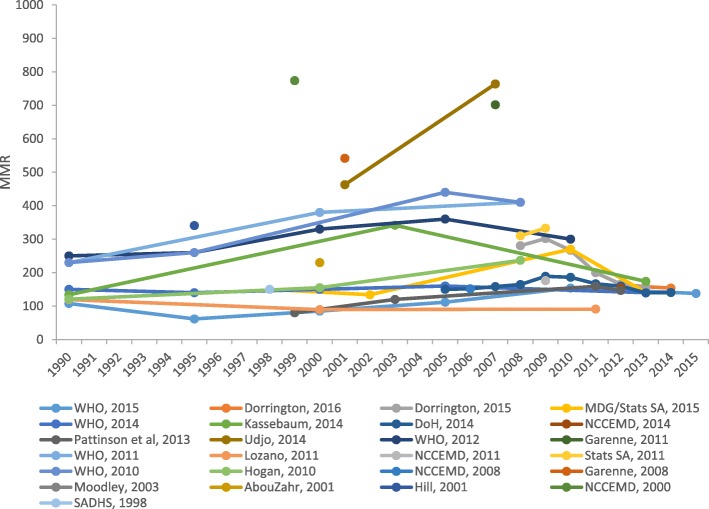
Trends in maternal mortality from 1990 to 2015 in South Africa

Figure 2 depicts the trend of maternal and mortality from 1990 to 2015. Estimates of MMR from most reports indicate an upward trend over time, at least until 2006 or 2009, thereafter a downward trend until 2015. Notably, four studies that ascertained maternal mortality using empirical [52, 54] and modelling [38, 65] approaches reported extreme estimates of MMR compared to other sources. Nevertheless, all recent estimates appeared to converge over time.

**Fig. 3:**
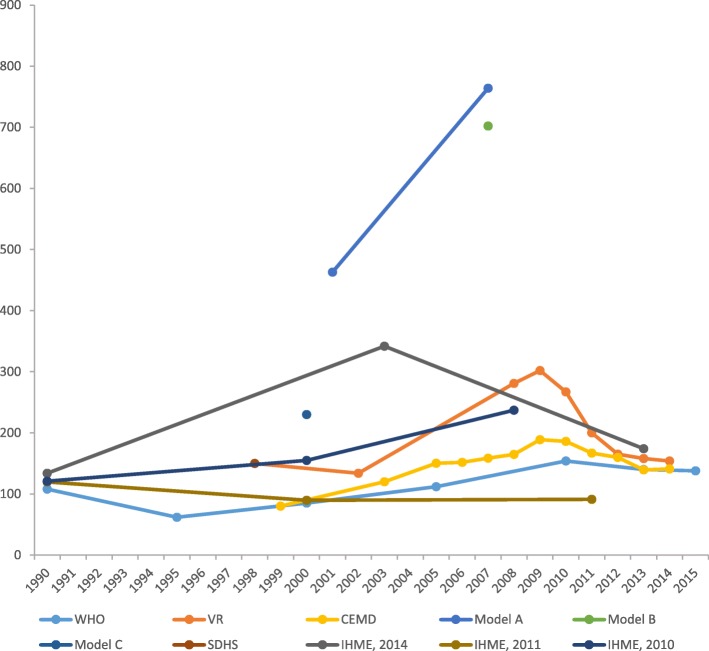
Trends in maternal mortality according to data source and estimation method. CEMD Confidential Enquiry to Maternal Deaths, IHME Institute for Health Metrics and Evaluation, SDHS South Africa Demographic and Health Survey, VR vital registration, WHO World Health Organization

Additionally, estimates of MMR reported by the global metrics (WHO) [68] were divergent from institutional reports (IHME) [41, 43, 69], and most modelled estimates (model A, B and C) [38, 65, 70] are widely divergent from estimates obtained through empirical methods (VR, CEMD and SDHS) [28, 45, 47–49, 51, 52, 55, 67]. The trend in MMR basing on estimates from confidential enquiry (CEMD) [28, 48, 49, 51, 53, 54, 67] and vital registration (VR) [45–47, 52] shows an increase until a maximum in 2009 followed by a drop in 2010. However, estimates from the vital registration and CEMD appeared to converge over time. Figure 3 shows trends in maternal mortality according to data source and estimation method using most up-to-date estimates superseding all previously published report.

#### Neonatal mortality

**Fig. 4:**
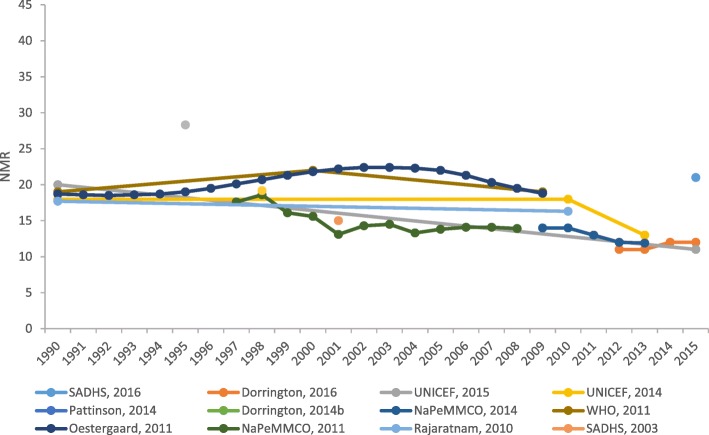
Trends in neonatal mortality from 1990 to 2015 in South Africa

Estimates of NMR from all sources indicate a slightly upward trend over time until 2004, followed by a steady decrease until 2013. Two single-year studies deriving their estimates using empirical [56] and modelling [64] approaches, respectively, reported substantially higher neonatal mortality rates than the others. Figure 4 depicts the trends of neonatal mortality from 1990 to 2015.

**Fig. 5:**
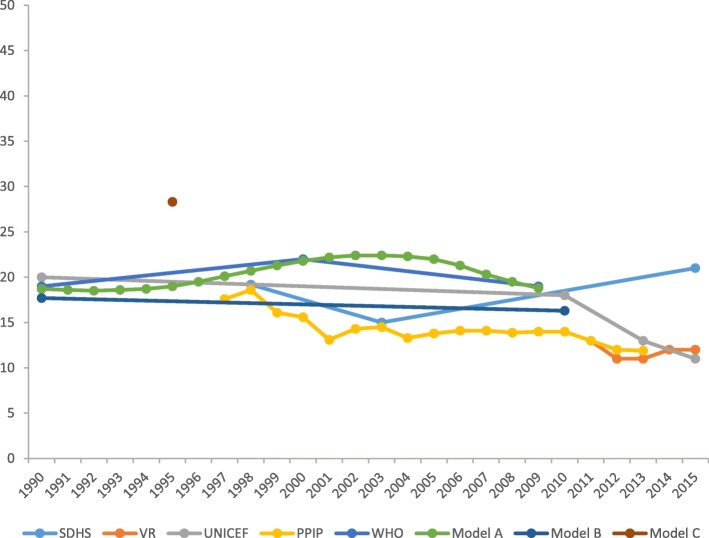
Trends in neonatal mortality according to data source and estimation method

Furthermore, estimates of NMR from global metrics (WHO and UNICEF) [14, 40, 61] were widely and periodically divergent from institutional reports (PPIP and CEMD) [23, 45, 57–59, 71]. Modelled estimates (WHO; UNICEF; model A, B and C) [14, 40, 61, 63, 64, 72] were large and divergent from estimates obtained through empirical methods (VR, PPIP and SDHS) [23, 45, 55–60, 71], with no clear pattern. The trend in NMR basing on estimates from the PPIP and vital registration shows a slight decline with periodic increase in neonatal mortality from 2000 to 2015 [23, 45, 57–59, 71]. Figure 5 shows trends in neonatal mortality according to data source and estimation method.

## Discussion

### Summary of evidence

This systematic review aimed to provide an overview of maternal and neonatal mortality from 1990 to 2015 for monitoring purposes, tracking progress and to advocate for resources and policy attention. In general, the estimates derived from all studies and reports indicated that South Africa did not achieve the MDG 4a and 5a goals of reducing under-five mortality by two thirds and maternal mortality by three quarters between 1990 and 2015, respectively. Despite the country struggling to achieve the MDG goals for maternal and neonatal mortality in the last two decades, recent reports showed significant progress made in reducing these outcomes [45, 68, 73].

### Broad trends

Although maternal and neonatal mortality are highly researched by both local and international authors or institutions in South Africa, there are considerable uncertainties around these estimates in the country. The possible reason for this might be high reliance on only a few data sources and limited empirical work. Accounting for the uncertainties about the actual levels of MMR and NMR in the country, estimates from both the institutional reports and global metrics indicated an upward trend in MMR and NMR until around 2006 and 2009. However, the increase in MMRs between 2001 and 2006 might specifically be explained by a consistent increase in HIV prevalence among pregnant women in the same period [74]. In addition, the downward trends in MMRs and NMRs from 2009 can be linked with the massive uptake of HIV treatment and an increased coverage of essential interventions, in particular the prevention of mother-to-child transmissions (PMTCT) of HIV which currently stands at over 90% [29, 30]. Nonetheless, all recent estimates are much more closely grouped indicating convergence over time.

### Challenges in measuring maternal and neonatal mortality

Evidence from the literature indicated that empirical methods, i.e. vital registration, household surveys and censuses, are subjected to misclassification and under-reporting of maternal deaths, thus leading to wide uncertainty intervals. Furthermore, estimating neonatal mortality from census and household surveys in high HIV prevalence settings is known to provide a biased estimate of child mortality due to correlations between HIV deaths in mother and death of her child [70].

### Highly variable estimates

Large margins of uncertainty associated with the estimated MMR and NMR highlight the need of interpreting these estimates with caution as well as not using them for monitoring trends over a short duration. The reasons for variations in the estimates of maternal and neonatal mortality remain poorly researched over the past two decades. In this review, we have observed a substantial discrepancy in the consistency of definitions used in the estimation of these outcomes, such as differentiating maternal deaths from pregnancy-related deaths [52, 65, 66]. Thus, uncertainties in estimates of MMR might be partly explained by differences in definitions used. Different estimation techniques used to obtain MMR and NMR necessitated the use of different data inputs, i.e. empirical data versus modelled estimates, which likely contributed to the divergent estimates of these outcomes.

For these reasons, cross-country comparisons, comparisons based on data from different sources and assessments of the overall burden become difficult. These comparisons should in many cases be interpreted with considerable caution due to different strategies being employed to derive such estimates. Evidence from recent studies focusing on estimating child mortality have revealed that methodological differences bias and compromise international comparisons of perinatal mortality [75–77]. Moreover, divergent estimates of MMR and NMR by different sources compromise interpretation of trends over time.

### Improving estimates

Over the past three decades, efforts have been made to improve the quality of maternal and neonatal mortality data due to the incompleteness of vital registration systems as well as the lack of reliable population surveys collecting detailed information on birth histories in the country. This included the introduction of modules about sibling history in national household surveys (e.g. Demographic and Health Survey (DHS)), including questions in censuses about whether a woman’s death was related to pregnancy, and the use of mixed methods cross-referencing facility records to determine the extent of under-registration of maternal deaths in vital registration system [4, 7, 78, 79]. However, these improvements could also have contributed to the increasing maternal and neonatal mortality over time.

Despite improvements in the completeness of death registrations in the last decade, the completeness of death registration has been reported to be lower in children as compared to adults and in rural areas than urban [79]. This might potentially explain some of the variability in estimates of both maternal and neonatal mortality in the country.

Generally, this review has revealed divergent estimates of MMR and NMR obtained from vital registration, household surveys, censuses and modelling over time. To obtain more accurate estimates, there is a need for applying additional estimation techniques which utilise available multiple data sources to correct for the underreporting of these outcomes, perhaps the capture-recapture method. This method is useful in resolving uncertainties in estimating conditions that have diverse estimates by operationalising statistically overlapping information from multiple data sources [80–84].

## Conclusions

Estimates from the global metrics and institutional reporting, although widely divergent, indicate South Africa has not achieved the MDG targets for maternal and neonatal mortality but made significant progress in reducing these outcomes in the last decade. Discrepancies in data sources and quality from which these estimates were obtained and highly variable estimates highlight the existence of uncertainties about the true estimates of maternal and child mortality in South Africa. In order to track progress and monitor the Sustainable Development Goals (SDGs) and the goal for health care for all by 2030, the country needs accurate, reliable, continuous and timely mortality statistics from the vital registration system, a clear understanding of any under-ascertainment of maternal or neonatal mortality and consistent approaches to accounting for these. It would be ideal if global agencies worked closely with local researchers to agree on the optimal calibration of South African estimates in multi-country models.

## Additional file

Additional file 1: Records of literature review search strategy. (DOCX 19 kb)
