# Supplementary material for: Trends in maternal and neonatal mortality in South Africa: a systematic review
Source: Syst Rev. 2019 Mar 27;8:76. doi: 10.1186/s13643-019-0991-y (PMC6436230; doi:10.1186/s13643-019-0991-y)
Supplement: Supplementary file 1 — Records of literature review search strategy. (DOCX 19 kb) [file 13643_2019_991_MOESM1_ESM.docx]

# Additional file 1: Records of literature review search strategy

| **Database** | **Search terms** | **Retrieved** | **Screened** | **Duplicate** |
| --- | --- | --- | --- | --- |
| **Medline** | (("mothers"[MeSH Terms] OR "mothers"[All Fields] OR "maternal"[All Fields]) OR ("infant, newborn"[MeSH Terms] OR ("infant"[All Fields] AND "newborn"[All Fields]) OR "newborn infant"[All Fields] OR "neonatal"[All Fields])) AND (("mortality"[Subheading] OR "mortality"[All Fields] OR "mortality"[MeSH Terms]) OR ("death"[MeSH Terms] OR "death"[All Fields])) AND (estimation[All Fields] OR estimates[All Fields]) AND ("South Africa"[Mesh] OR ("south africa"[MeSH Terms] OR ("south"[All Fields] AND "africa"[All Fields]) OR "south africa"[All Fields])) AND (("1990/01/01"[PDAT] : "3000/12/31"[PDAT]) AND "humans"[MeSH Terms] AND English[lang]) | 168 | 110 | 58 |
|  | **Limits:**  English language  Year: 1990-2017  Study on humans |  |  |  |
| **Africa-wide Information** | (Maternal OR mother OR neonatal OR infant OR newborn) AND (mortality OR death) AND estimat* AND South Africa | 248 | 118 | 130 |
|  | **Limits:**  English language  Year: 1990-2017 |  |  |  |
| **Web of Science** | (Maternal OR mother OR neonatal OR infant OR newborn) AND (mortality OR death) AND estimat* AND South Africa | 257 | 168 | 89 |
|  | **Limits:**  English language  Year: 2005-2017 |  |  |  |
| **Cinahl** | (Maternal OR mother OR neonatal OR infant OR newborn) AND (mortality OR death) AND estimat* AND South Africa | 52 | 30 | 22 |
|  | **Limits:**  English language  Year: 1990-2017 |  |  |  |
| **Scopus** | (Maternal OR mother OR neonatal OR infant OR newborn) AND (mortality OR death) AND estimat* AND “South Africa” | 223 | 223 | 0 |
|  | **Limits:**  English language  Year: 1990-2017 |  |  |  |
| **Extra** | **Reference collected from bibliography of key articles** | 21 | 21 | 0 |
| **Total** | **Total unique reference** | **969** | **670** | **299** |
